# Polyphenolic compounds of amla prevent oxidative stress and fibrosis in the kidney and heart of 2K1C rats

**DOI:** 10.1002/fsn3.1640

**Published:** 2020-05-20

**Authors:** Md. Mizanur Rahman, Khandoker Usran Ferdous, Shraboni Roy, Iffat Ara Nitul, Fariha Mamun, Md. Hemayet Hossain, Nusrat Subhan, Md Ashraful Alam, Md. Areeful Haque

**Affiliations:** ^1^ Department of Pharmaceutical Sciences North South University Dhaka Bangladesh; ^2^ BCSIR Laboratories Bangladesh Council of Scientific and Industrial Research Dhaka Bangladesh; ^3^ Department of Pharmacy International Islamic University Chittagong Chittagong Bangladesh; ^4^ Drug and Herbal Research Centre, Faculty of Pharmacy Universiti Kebangsaan Malaysia Kuala Lumpur Malaysia

**Keywords:** Amla, antioxidant, *Emblica officinalis*, fibrosis, oxidative stress

## Abstract

Amla (*Emblica officinalis* Gaertn.) is a natural source of antioxidants and possesses valuable medicinal properties. However, the protective effect of amla in the kidney of two‐kidneys‐one‐clip (2K1C) rats has not been explained sufficiently. This study was performed to evaluate the renoprotective effect of amla fruit powder (2.5% W/W) supplementation in kidneys of 2K1C rats. 2K1C rats increased the remnant kidney wet weight and also increased plasma creatinine and uric acid concentration compared to the control. Amla supplementation ameliorates elevated creatinine and uric acid concentration in plasma of 2K1C rats. Various oxidative stress indicators such as malondialdehyde, nitric oxide (NO), and advanced protein oxidation product (APOP) were also increased in plasma, heart, and kidney tissues in 2K1C rats that were also significantly brought down to normal level by amla supplementation. Moreover, the inflammatory cells entry and fibrosis in the 2K1C rat's tissues were prevented by amla supplementation. These research results suggest that amla may restore plasma antioxidant capacities and prevents oxidative stress, inflammation, and fibrosis in 2K1C rats. Taken these results as a base, clinical supplementation of dried amla powder in diet or juice to the CKD patients would be beneficial.

## Graphical abstract

HPLC analysis of amla revealed the presence of polyphenolic antioxidants. Amla markedly reduced the levels of oxidative stress markers in 2K1C rats. Amla improved the levels of endogenous antioxidant enzymes, namely, CAT and SOD. Amla reduced the plasma levels of uric acid and creatinine in 2K1C rats.

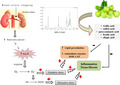

## 1 INTRODUCTION

Kidney dysfunction and cardiac fibrosis have become one of the rising problems in the last few years in large populations worldwide. Kidneys serve several essential regulatory roles in the purification of body fluid and controlling blood pressure in the human body. Their main operation is to regulate electrolytes balance and control pH in blood (Johnson, 1985). Additionally, kidneys are involved in filtering the waste materials including urea, nitrogenous products, drug metabolites, and maintain body's fluid balance. Kidneys also release hormones such as renin which may participate in the regulation of blood pressure (Rudroju, Schukat, & Kidd, 2011). However, the prevalence of kidney dysfunction is increasing dramatically, and unfortunately, the cost of treating kidney dysfunction has become enormous burden on healthcare systems worldwide (Yodchai, Dunning, Savage, & Hutchinson, 2016). Hypertension, increased uric acid content, diabetes, and inflammation are several factors, which induce chronic renal failure (Alicic, Rooney, & Tuttle, 2017; Sharaf El Din, Salem, & Abdulazim, 2017). Renin–angiotensin system (RAS) activation also plays a critical role in the development of hypertension and chronic kidney diseases (Mullins, Conway, Menzies, Denby, & Mullins, 2016). Kidney injuries may occur due to excessive oxidative stress to tissues and also by increasing the free radical generation and decreasing antioxidant defense (Dennis & Witting, 2017; Himmelfarb et al., 2004). Earlier studies showed that antioxidant therapy would have benefits in reducing the risk of cardiovascular disease in people with chronic kidney disease (CKD) (Tamadon, Zahmatkesh, & Mousavi, 2015). In the two‐kidneys‐one‐clip model (2K1C), a clip is placed on the renal artery of the left kidney (Mullins et al., 2016). The 2K1C model is similar to the volume overload form of hypertension, and rats are generally used in this animal model for well understanding of angiotensin II‐mediated hypertension and related complications in kidney and hearts (Ding et al., 2020).

*Emblica officinalis* Gaertn. (Amla or Indian gooseberry) has been described in many prolific types of research to possess multiple therapeutic activities and is considered as an essential part of many formularies of Ayurveda and Unani medicines as well (Gopa, Bhatt, & Hemavathi, 2012). Earlier literature reports illustrate that amla possesses antioxidant (Bhattacharya, Chatterjee, Ghosal, & Bhattacharya, 1999), hepatoprotective (Damodara Reddy, Padmavathi, Gopi, Paramahamsa, & Varadacharyulu, 2010), nephroprotective (Yokozawa et al., 2007), hypolipidemic (Kim, Okubo, Juneja, & Yokozawa, 2010), cardioprotective (Ojha, Golechha, Kumari, & Arya, 2012), and antidiabetic effects (Ansari et al., 2014) in various animal models. Additionally, amino acids, vitamin C, and minerals are found in amla. Tannins, rutin, emblicol, phyllembelic acid, phyllemblin, and curcuminoids are some of the secondary metabolites also present in amla (Zhang, Tanaka, Iwamoto, Yang, & Kouno, 2000). Furthermore, amla has been proven to be a prominent source of phenolic compounds including phenolic acids and flavonoids (G. S. Kumar, Nayaka, Dharmesh, & Salimath, 2006). Previous studies reported that amla extract is beneficial in preventing acute kidney dysfunction (Tasanarong, Kongkham, & Itharat, 2014) and age‐related renal dysfunction (Yokozawa et al., 2007). By considering these, it can be expected that amla powder would prevent free radical‐mediated oxidative stress and may improve related health disorder. Hence, this study was conducted to evaluate the therapeutic effect of amla in ameliorating the oxidative stress in heart and kidney dysfunction in 2K1C rats.

## 2 MATERIALS AND METHODS

### 2.1 Chemicals & reagents

Gallic acid (GA), ellagic acid (EA), vanillin (VL), vanillic acid (VA), caffeic acid (CA), pyrogallol (PG), (+)‐catechin hydrate (CH), (‐)‐epicatechin (EC), kaempferol (KF) p‐coumaric acid (PCA), rosmarinic acid (RA), rutin hydrate (RH), myricetin (MC), and quercetin hydrate (QU) used in this study were bought from Sigma‐Aldrich (St. Louis, MO, USA). Acetonitrile (HPLC), acetic acid (HPLC), methanol (HPLC), and ethanol were procured from Merck (Darmstadt, Germany).

### 2.2 Amla fruit collection, identification, and supplement preparation

Amla fruit was purchased from the local market of, Bangladesh, which was authenticated by Dr. Md Ashraful Alam, Associate Professor, Department of Pharmaceutical Sciences, North South University. The fruits were shade‐dried and then ground into coarse particles using a grinding machine. This coarse powder of amla (2.5% w/w) was mixed properly with powdered chow food and used as supplementation in this study. The ethanol crude extract of amla fruit powder was prepared by maceration of the powder with ethanol, and then, the solvent was allowed to get evaporated using a rotary evaporator to finally prepare the crude extract to be used for phenolic content analysis by HPLC.

### 2.3 Phenolic content determination in amla extract by HPLC‐DAD system

The phenolic composition of the ethanol extract of amla was determined by HPLC (Dionex UltiMate 3,000 system from Thermo Scientific, having quaternary rapid separation pump (LPG‐3400RS) and photodiode array detector (DAD‐3000RS)), as described previously. Mobile phase solvent system was prepared by using acetonitrile (solvent A), acetic acid (pH 3.0, solvent B), and methanol (solvent C). The detection and quantification of gallic acid (GA), catechin hydrate ( CH), epicatechin (EC), vanillic acid (VA), and caffeic acid (CA) were done at 280 nm, of *p*‐coumaric acid (PCA), ellagic acid (EA), and rutin hydrate (RH) at 320 nm, and of myricetin (MC), quercetin (QU), and kaempferol (KF) at 380 nm, respectively. A standard stock solution of each phenolic compound (100 µg/ml) was prepared in methanol. A solution of ethanol extract of amla at a concentration of 5 mg/ml was prepared in ethanol by vortex mixing (Branson, USA) for 30 min.

### 2.4 Experimental animals design and diet

From the Animal House at the Department of Pharmaceutical Sciences, North South University, twenty‐eight male Long Evans rats, weighing between 180 and 210 g and aging from ten to twelve weeks old, were procured for the purpose of this study. They were kept in plastic cages with one rat per cage. The room temperature was maintained at 25 ± 3°C. A 12 hr dark/light cycles were also maintained in every day. Regular laboratory diet and water were provided to all rats. The Ethical Committee of North South University reviewed and granted all experimental protocols. All the animals were randomly distributed into four experimental groups (seven rats in each group) as follows:

**Control** rats were given powdered chow food and water only;

**Control + Amla** rats were provided with powdered chow food with supplemented amla powder (2.5% of powdered chow food, w/w) and water every day for four weeks;

**2K1C** underwent surgery and clipping in one renal artery and supplemented with 1% sodium chloride mixed in their drinking water and also given powdered chow food;

**2K1C + Amla** underwent surgery and clipping in one renal artery and supplemented with 1% sodium chloride mixed in their drinking water along with amla powder with powdered chow food (2.5% of powdered food, w/w) every day for four weeks.

Laboratory chow food was used in this study. The chow food are composed of wheat, wheat bran, rice polishing, and fish meal, having calories as percentage, for example, 25% proteins, 60% carbohydrates, and 15% fat approximately **(**Table 1).

**Table 1:** Compositions of normal diet used in this study for 100 g

Control diet	Percentage (%)
Wheat	40%
Wheat bran	20%
Rice polishing	0.5%
Fish meal	1.0%
Oil cake	1.0%
Gram	0.39%
Pulses	0.39%
Milk	0.38%
Soybean oil	0.15%
Molasses	0.095%
Salt	0.095%
Embavit (vitamin mixture)	0.1%

### 2.5 Animal sacrifice and preparation of tissue samples.

Following the end of the treatment period of 28 days, all animals were weighed. They were sacrificed using a high dose of pentobarbitone anesthesia (75 mg/kg) followed by their blood collection from an abdominal vein with an 18 gauge needle and placement in tubes containing citrate buffer. The collected blood was separated by centrifuge at 7000 g, and the plasma was then preserved in a refrigerator at −20°C for the next level of analysis. All the organs collected were wet weighed, and one part of the heart and both kidneys of the rats from all groups were stored in neutral buffered formalin (NBF, pH 7.4) for histological evaluation immediately after weighing whereas another part of the tissues was placed in the refrigerator at −20°C just as the serum for the biochemical test. The preserved, refrigerated kidney and heart tissues were liquidified homogenously using phosphate buffer of pH 7.4 and then centrifuged at 7000 g for 15 min at 4ºC following which the supernatant was separated and finally utilized in the protein and enzymatic studies as has been described below.

### 2.6 Assessment of oxidative stress markers

Lipid peroxidation in tissues was analyzed calorimetrically following a previously conducted work (Niehaus Jr & Samuelsson, 1968), and at the wavelength of 532 nm, the sample absorbance was determined against a reference blank solution. NO was estimated as nitrate by using a standard curve according to a method described before (Tracey, Tse, & Carter, 1995) and was expressed as nmol/gm of tissue. Furthermore, APOP level was measured using the method of Witko‐Sarsat et al. (1996) (Witko‐Sarsat et al., 1996).

### 2.7 Determination of antioxidant activities

Superoxide dismutase (SOD) level in tissue homogenates was evaluated by following a method described previously by Tripathy and Chandra (2010) (Tripathi & Chandra, 2010). One unit enzyme activity was evidenced from 50% reduction of the auto‐oxidation of epinephrine contained in the sample solution. Catalase activity assay protocol was described previously (Khan, 2012). An absorbance alteration of 0.01 was considered as a unit activity of CAT that was indicated as units/min.

### 2.8 MPO activity estimation

A modified dianisidine‐H_2_O_2_ method was utilized to estimate the level of a tissue inflammation marker, myeloperoxidase (MPO) (Ulla et al., 2017). Briefly, samples (10 µg of protein as tissue homogenate) were added in triplicate to the mixture of *o*‐dianisidine dihydrochloride (0.53 mM) and H_2_O_2_ (0.15 mM) in PBS followed by measurement of alterations in the reaction mixture's absorbance at 460 nm. MPO activity was expressed as MPO/mg protein.

### 2.9 Evaluation of liver and kidney function markers

Within 30 min of transferring into citrate buffer‐containing tubes, the blood was centrifuged at 7000 g for 10 min at 4°C and processed for biochemical analysis. Plasma levels of alanine aminotransferase (ALT), aspartate aminotransferase (AST), alkaline phosphatase (ALP) activities and kidney function markers; that is, creatinine and uric acid were measured according to manufacturer's instructions (DCI Diagnostics, Hungary).

### 2.10 Histopathological examination

Neutral buffered formalin (10%) was used to fix the kidney and heart tissues for histological analysis, which was also changed several times for better processing of the tissues. These tissues were further processed with graded ethanol and xylene treatments. The processed tissue samples were put into paraffin pieces carefully in order to avoid air entry. These paraffin blocks‐containing tissues were then sectioned into slices of about five *µ*m thickness using a rotary microtome machine. Following the slicing, the tissue slices were placed on clean glass slides gently and then stained with hematoxylin and eosin (H&E) following routine procedures to observe tissue inflammation. Picrosirius red staining was performed to detect the fibrosis in the renal and left ventricular tissues. Stained tissue sections were then thoroughly analyzed for any morphological changes, and their photographs were taken under a light microscope (Zeiss Axio Scope) at X40 magnifications.

### 2.11 Statistical analysis

All data have been presented as mean ± *SEM*. By utilizing GraphPad prism software, Newman–Keul's test was performed following One‐way ANOVA for statistical evaluation. In this study, statistical findings were expected to be significant in every case with a *p*‐value of less than .05.

## 3 RESULTS

### 3.1 Phenolic content analysis in amla extract

**Figure 1:**
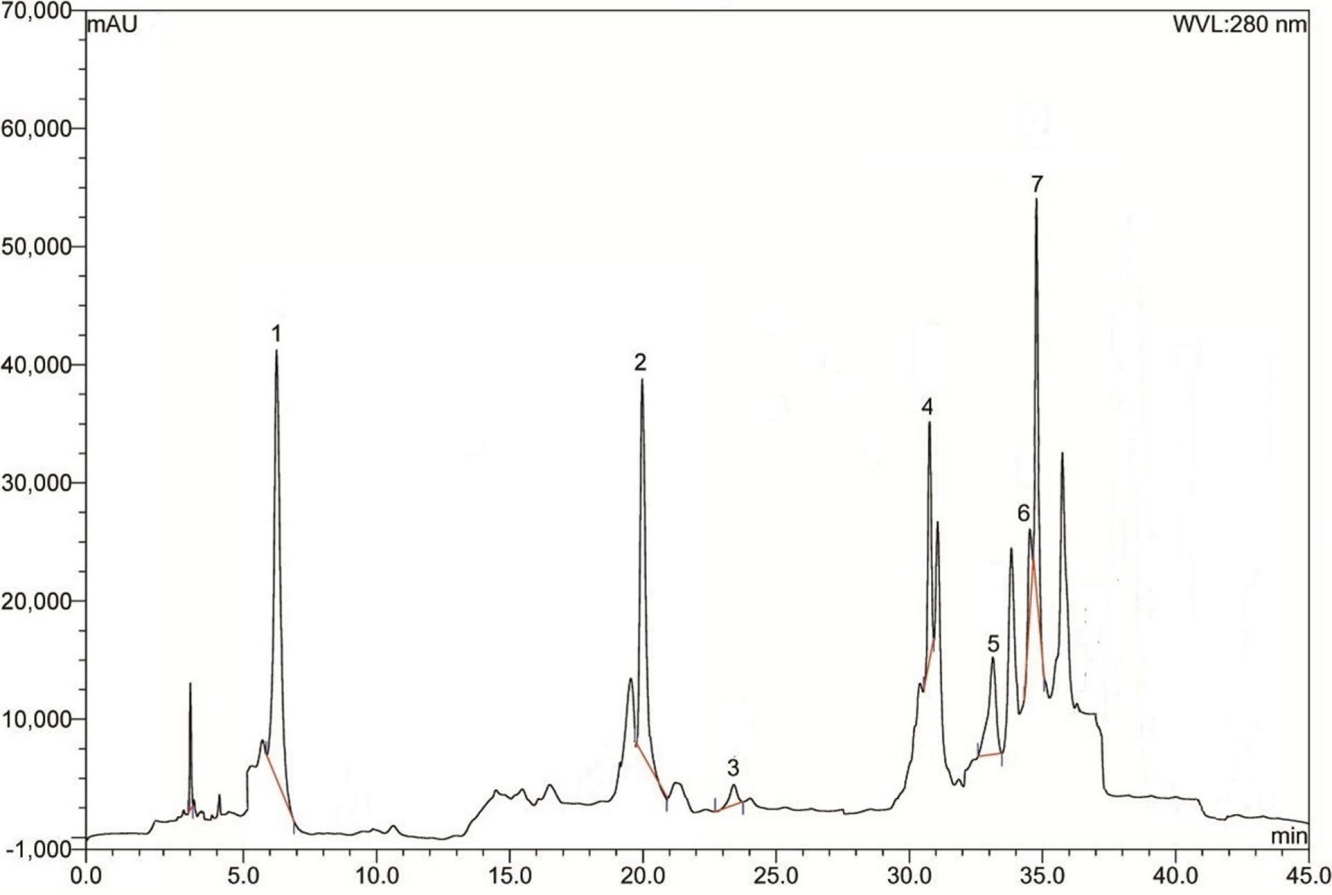
HPLC chromatogram of alcoholic extract of amla. Peaks: 1, gallic acid (GA); 2, (+) catechin hydrate (CH); 3, caffeic acid (CA); 4, *p*‐coumaric acid (PCA); 5, *trans*ferulic acid (FA); 6, rutin hydrate (RH); 7, ellagic acid (EA)

The presence of phenolic antioxidants in the ethanol extract of amla was evaluated by HPLC‐DAD system. The HPLC chromatogram is presented in Figure 1. Gallic acid, caffeic acid, para‐coumaric acid, ferulic acid, and ellagic acid were found in good amount, and the amounts present in the extract are given in Table 2.

**Table 2:** Contents of polyphenolic compounds in the alcoholic extract of amla (*n* = 5)

Polyphenolic compound	Alcoholic extract of amla	
	Content (mg/100 g of dry extract)	% RSD
Gallic acid	65.12	1.17
Catechin hydrate	55.03	1.14
Caffeic acid	9.77	0.05
para‐caumaric acid	30.81	0.86
Ferulic acid	28.35	0.73
Rutin hydrate	5.67	0.04
Ellagic acid	74.83	1.39

RSD, relative standard deviation.

### 3.2 Effect of amla on body weight, food intake, water intake, and organ weights of 2K1C rats

In this study, rats that underwent 2K1C surgery showed decreased body weight and reduced food and water consumption than the control rats and treatment group increased body weight, food intake, and also water intake (Table 2). 2K1C rats showed an increase in the right kidney wet weight compared to the control rats (*p* < .05). However, no reduction of the right kidney wet weight was noticed in the amla powder supplemented 2K1C rats (Table 3). Furthermore, heart wet weights remain almost unchanged among the group tested (Table 3).

**Table 3:** Effects of amla powder supplementation on body weight, remnant kidney wet weight, and heart wet weight of 2K1C rats

Parameters	Control	2K1C	Control + Amla	2K1C + Amla
Initial body weight	176.67 ± 3.65_a_	204.95 ± 1.62_a_	195.96 ± 3.82_a_	182.51 ± 5.85_a_
Final body weight	228.78 ± 5.16_a_	211.98 ± 7.57_b_	259.04 ± 3.61_c_	255.04 ± 5.75_c_
Food intake (g)	22.67 ± 1.10_a_	19.20 ± 1.21_b_	23.89 ± 0.73_c_	22.91 ± 0.72_a_
Water intake (g)	22.21 ± 2.02_a_	18.92 ± 0.82_b_	24.90 ± 0.87_c_	21.09 ± 0.97_a_
Kidneys wet weight	0.30 ± 0.01_a_	0.42 ± 0.01_b_	0.30 ± 0.01_a_	0.45 ± 0.02_a,c_
Heart wet weight	0.24 ± 0.01_a_	0.28 ± 0.01_b_	0.24 ± 0.012_a_	0.26 ± 0.011_c_

Note

Statistical analysis was done by one‐way ANOVA followed by Newman–Keuls post hoc test. Statistical significance was considered as *p* < .05 and marked with an asterisk symbol; here, _a_ versus _b_ is significantly different at *p* < .05 level and _b_ versus _c_ significantly different at *p* < .05. All other nonmarked groups are not significantly different.

### 3.3 Effect of amla on plasma AST, ALT, and ALP levels in 2K1C rats

**Figure 2:**
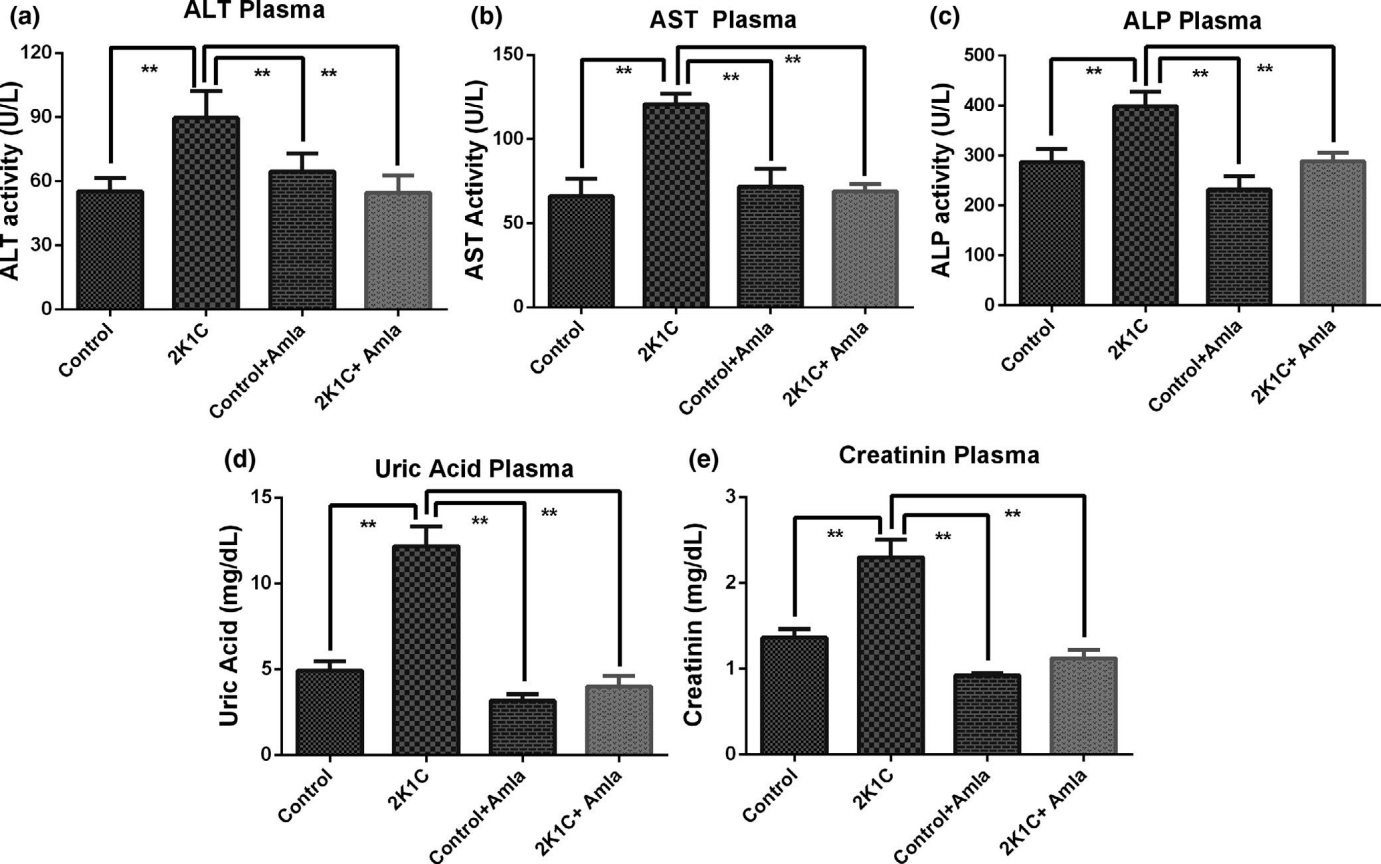
Effect of amla powder supplementation on ALT, AST, ALP activities, and uric acid and creatinine concentrations in plasma of 2K1C rats. Data are expressed as mean ± *SEM*, *n* = 7. Statistical analysis was done by one‐way ANOVA followed by Newman–Keuls post hoc test. Statistical significance was considered as *p* < .05 and marked with an asterisk symbol

The ALT and AST levels were increased prominently (*p* < .05) in the 2K1C rats in comparison with both control rats and control + amla rats (Figure 2a,2b). Amla treatment of 2K1C rats helped normalize their elevated ALT and AST enzymes activities significantly (*p* < .05) compared to the untreated, 2K1C rats (Figure 2a,b). Likewise, ALP activity was also found to be remarkably (*p* < .05) higher in 2K1C rats than both control rats and control + amla rats and this increase in ALP enzyme level was prevented significantly (*p* < .05) by amla treatment (Figure 2c). In addition, levels of these three enzymes in the control rats treated with amla powder were also close to their respective levels of the untreated, control rats (Figure 2a,b,c).

### 3.4 Effect of amla on uric acid and creatinine concentrations in serum of 2K1C rats

Uric acid and creatinine are two important markers of kidney function. In this study, 2K1C rats showed a notably (*p* < .05) increased uric acid concentration compared to the control group. Amla powder supplementation in 2K1C rats significantly (*p* < .05) ameliorated the elevated uric acid level in their serum compared to the diseased, 2K1C rats (Figure 2d). On the other hand, creatinine level in the serum of 2K1C rats was also increased significantly (*p* < .05) compared to the control group which was markedly (*p* < .05) brought down close to normal level by amla powder supplementation (Figure 2e). Moreover, in both cases of uric acid and creatinine, the control rats supplemented with amla powder displayed significantly (*p* < .05) reduced levels of these two markers in contrast with the 2K1C rats and these values were also slightly lower than that of the control rats (Figure 2d,e).

### 3.5 Effect of amla on oxidative stress markers in plasma, kidney, and heart tissue homogenates of 2K1C rats

**Figure 3:**
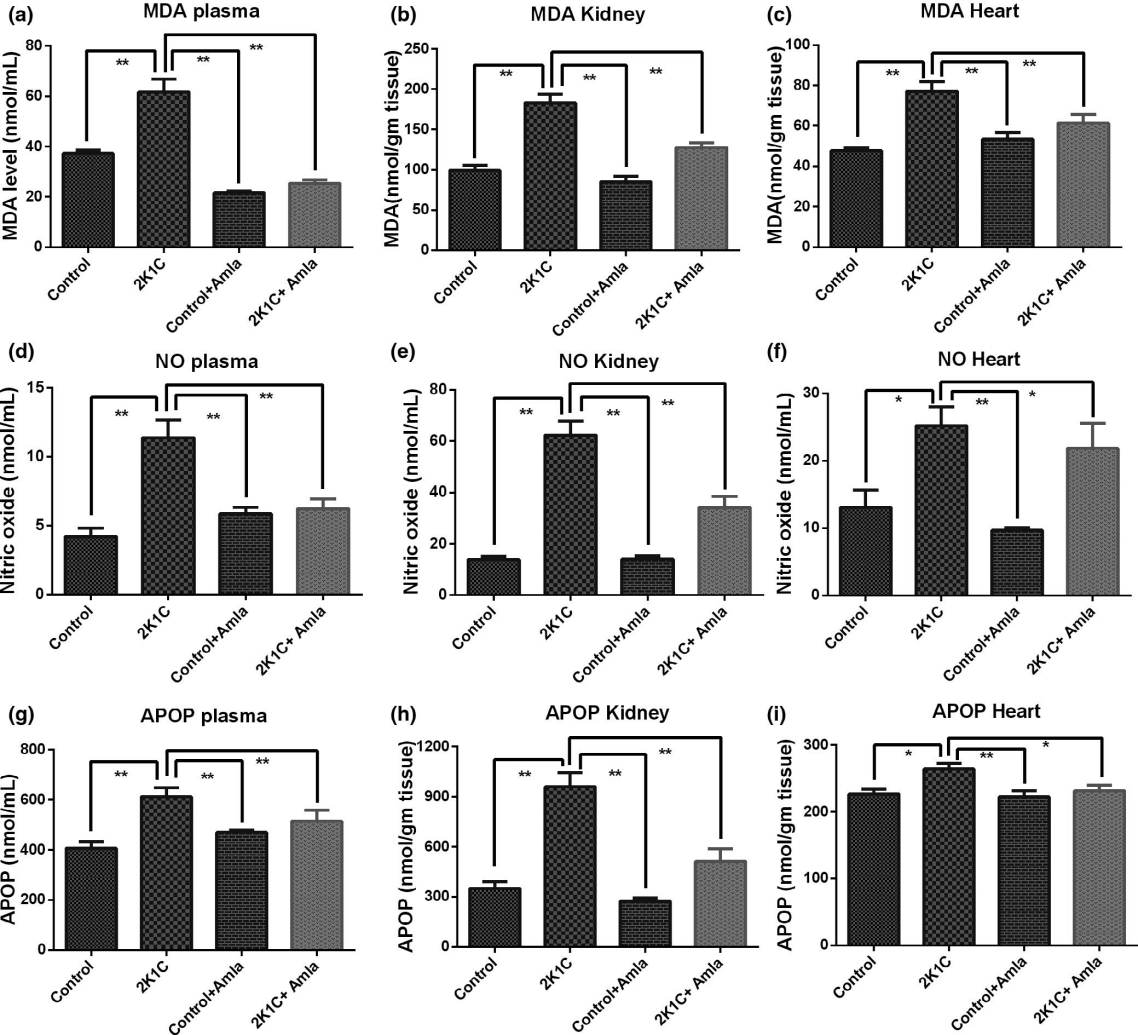
Effect of amla powder supplementation on the oxidative stress parameters MDA, NO, and APOP in plasma, heart, and kidney tissue homogenates of 2K1C rats. Data are expressed as mean ± *SEM*, *n* = 7. Statistical analysis was done by one‐way ANOVA followed by Newman–Keuls post hoc test. Statistical significance was considered as *p* < .05 and marked with an asterisk symbol

This current investigation indicated that oxidative stress parameters were increased in plasma, heart, and kidney of the 2K1C rats compared to the control group. The most salient oxidative stress parameter is the malondialdehyde (MDA), a lipid peroxidation product. 2K1C rats showed significantly (*p* < .05) increased MDA level in plasma, heart, and kidney tissue homogenates compared to the control rats and the control + amla rats which is a clear indication of lipid peroxidation in cell membranes. However, amla powder supplementation in 2K1C rats decreased or normalized the elevated MDA formation in plasma, kidney, and heart tissue homogenates remarkably (*p* < .05) compared to the untreated, 2K1C rats (Figure 3a–c). Moreover, this study demonstrates that nitric oxide level has been found to be significantly (*p* < .05) higher in plasma and kidney tissue homogenate of 2K1C rats than both the control rats and the control + amla rats. Amla powder supplementation markedly (*p* < .05) reduced the serum and kidney tissue levels of nitrite/nitrate in 2K1C rats in comparison to the diseased rats (Figure 3d,e). Furthermore, the advanced protein oxidation product (APOP) is an essential oxidative stress marker in tissues which might be formed due to the increased chlorinated free radicals reacted with plasma proteins. According to this investigation, APOP concentrations were got significantly (*p* < .05) elevated especially in the plasma and kidney tissue homogenate of 2K1C rats than both amla‐treated and untreated control rats which were found to be significantly (*p* < .05) decreased in those 2K1C rats supplemented with amla powder (Figure 3g,h). Nevertheless, no profound difference in the levels of these three oxidative stress markers was found between the two groups of amla powder‐treated and untreated rats (Figure 3a–i).

### 3.6 Effect of amla on antioxidant enzyme activity in plasma, kidney, and heart tissue homogenates of 2K1C rats

**Figure 4:**
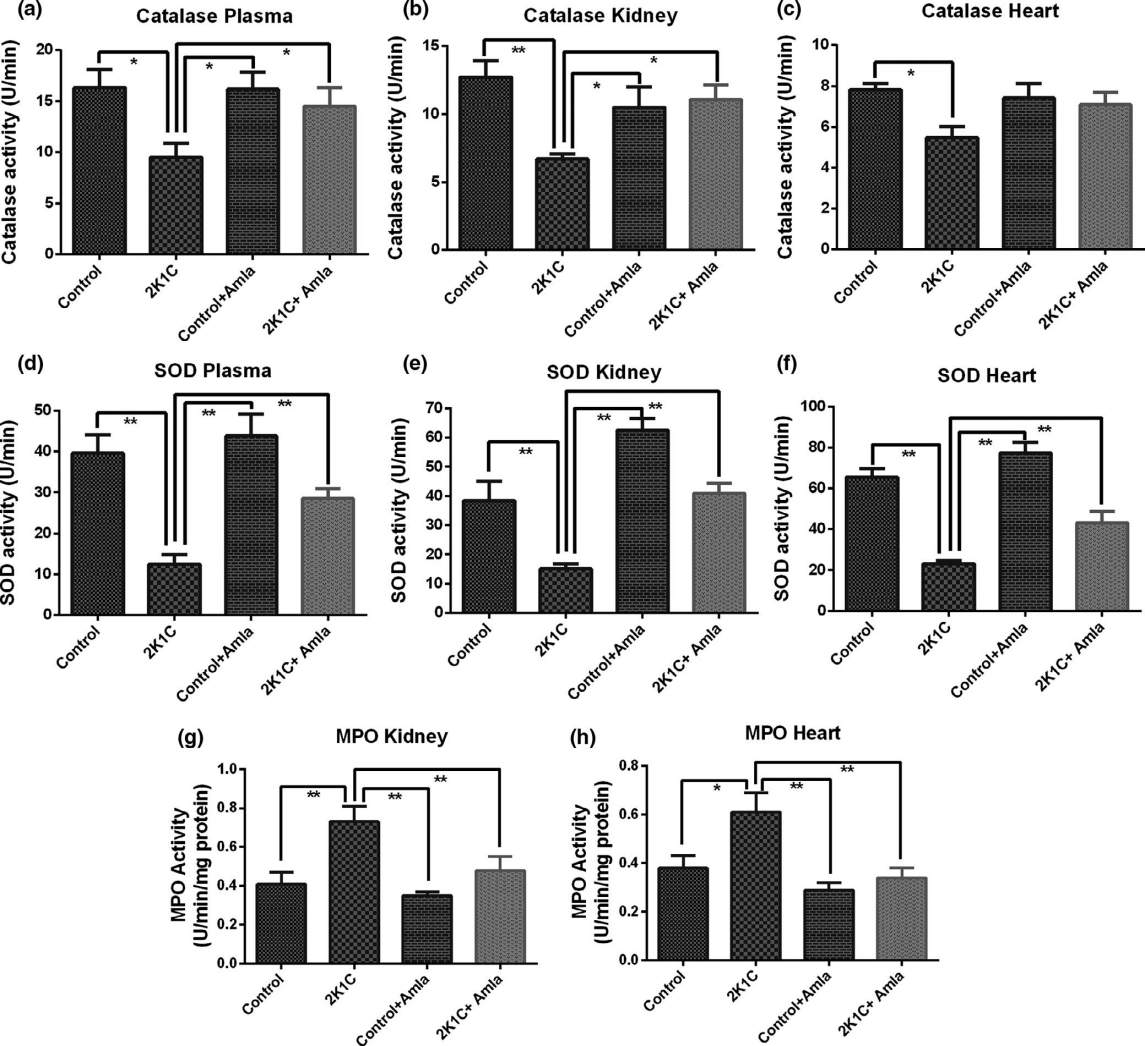
Effect of amla powder supplementation on antioxidant enzyme catalase **and SOD** activities in plasma, heart, and **kidney** tissue homogenates and MPO concentration in kidney and heart tissue homogenates of 2K1C rats. Data are expressed as mean ± *SEM*, *n* = 7. Statistical analysis was done by one‐way ANOVA followed by Newman–Keuls post hoc test. Statistical significance was considered as *p* > .05 and marked with an asterisk symbol

Compared to both amla powder‐treated and untreated control rats, the levels of both catalase and SOD antioxidant enzymes were found to get significantly (*p* < .05) declined in the plasma and kidney tissue homogenate of the diseased, 2K1C rats (Figure 4a,b,d,e) with such pronounced reduction also found in the heart tissue homogenate by SOD (Figure 4f). This study shows that the diminished catalase activity in the plasma and kidney tissue homogenate of 2K1C rats was enhanced by amla powder supplementation in comparison with the diseased, 2K1C rats with no remarkable effect found on the heart tissue homogenate (Figure 4a–c). However, amla powder supplementation effectively (*p* < .05) restored SOD activity in the plasma, kidney, and heart tissue homogenates of 2K1C rats in comparison with the untreated, 2K1C rats (Figure 4d–f). Overall, amla powder treatment had more prominent effect in case of SOD than in catalase.

In addition, MPO activity was increased in kidney and heart tissue homogenates of 2K1C rats with significant (*p* < .05) increment noticed especially in case of kidney tissue compared to the control rats. However, the elevated MPO levels in both kidney and heart tissues were significantly (*p* < .05) brought down to near normal level by amla powder supplementation in 2K1C rats (Figure 4g,h). On the other hand, amla powder‐treated control rats showed almost similar level of MPO in kidney and heart as in the normal, control rats (Figure 4g,h).

### 3.7 Histological assessment of the effect of amla powder on kidney and heart tissues of 2K1C rats

**Figure 5:**
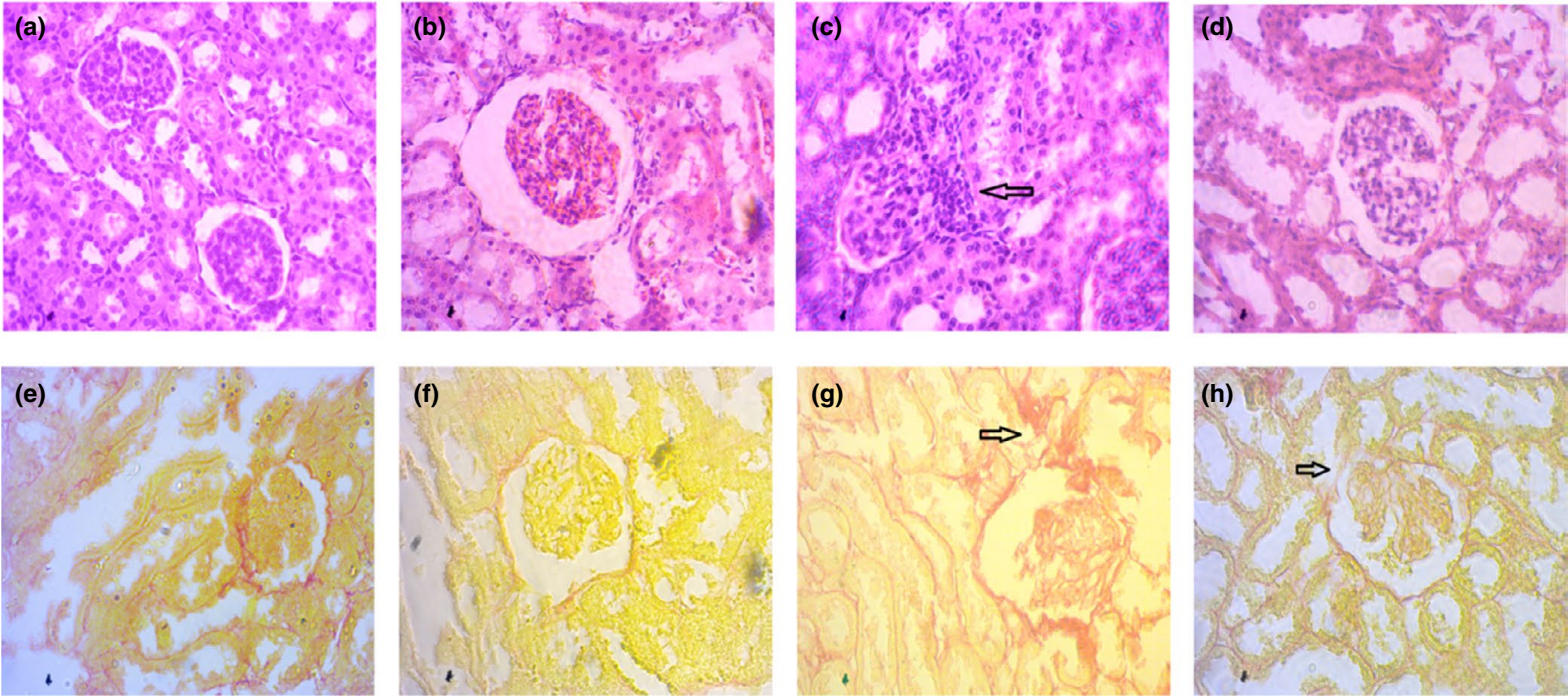
Effect of amla powder supplementation on renal inflammation and fibrosis in the kidney of 2K1C rats. a, e—control, showed normal histological structure in kidney section of rats; b, f—Control+Amla, also showed normal histological structure in kidney section of rats; c, g—2K1C, showed inflammatory cells infiltration and fibrosis in kidney section of 2K1C rats; and d, h—2K1C+Amla, amla supplementation prevented the inflammatory cells infiltration and fibrosis in the kidney of 2K1C rats

Both H&E‐stained and Picrosirius red‐stained kidney sections revealed normal glomerular structure in case of both amla powder‐untreated and treated control rats (Figure 5a,e,b,f) which were found to get shrinked and infiltrated with inflammatory cells in case of 2K1C rats (Figure 5c). 2K1C rats also showed tubular wall damage and cellular necrosis in contrast with the control rats (Figure 5c). Moreover, 2K1C rats exhibited visible fibrosis and collagen deposition in the kidney compared to the control and control + amla rats (Figure 5g). It was observed that amla powder supplementation in 2K1C rats helped in improving the distorted kidney structure along with reduction of inflammatory cells entry and fibrotic damages (Figure 5d,h).

**Figure 6:**
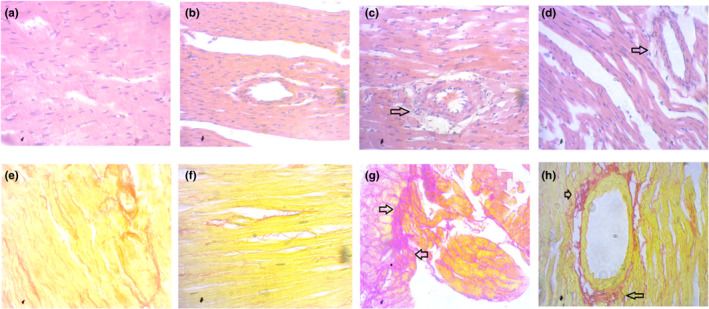
Effect of amla powder supplementation on cardiac inflammation and fibrosis in the heart of 2K1C rats. a, e—control, showed normal histological structure in heart section of rats; b, f—Control+Amla, also showed normal histological structure in heart section in rats; c, g—2K1C, showed tissue scar and collagen deposition as fibrosis in heart section in 2K1C rats; d, h—2K1C+Amla, amla supplementation prevented the heart tissue from damage and reduced collagen deposition in 2K1C rats

On the other hand, normal histoarchitecture was noticed in both types of stained heart tissues of control and control + amla rats as well (Figure 6a,e,b,f) 2K1C rats displayed necrotic changes in the H&E‐stained left ventricle of the heart as well as a surge of inflammatory cells compared to the control and control + amla rats (Figure 6c). The cardiac tissue necrosis and inflammatory cells entries were reduced by amla supplementation in 2K1C rats (Figure 6d). Moreover, massive fibrosis and collagen deposition were also observed in Picrosirius red‐stained 2K1C rat's heart in comparison to the control rats (Figure 6g). Amla supplementation visibly ameliorated the fibrosis and excessive collagen deposition in the left ventricle of the heart of 2K1C rats (Figure 6h).

## 4 DISCUSSION

The present work demonstrates the protective effects of amla powder on kidney dysfunction in 2K1C rats that also helps in preventing lipid peroxidation and fibrosis. These preventive effects could be attributed to amla powder's high capacity of scavenging free radicals in tissues. The 2K1C surgery‐induced kidney injury leads to the subsequent development of oxidative stress and fibrosis and for decades, 2K1C model has been used as a suitable animal model for kidney disease research (Ding et al., 2020). It has been postulated that oxidative stress plays a major role in the molecular mechanism involved in the experimental kidney damage of such animal models. Following 2K1C surgery, less blood supply to one kidney triggers the activation of renin–angiotensin system and ultimately causes the development of oxidative stress in renal system by promoting reactive oxygen species (ROS) production (Investigators, 2014). These excessively generated ROS oxidize cellular biomolecules and then carry out oxidation of membrane phospholipids. Cardiomyocytes and blood vessels are mostly affected by such ROS‐induced oxidation. Free radicals generated in tissues can disrupt the cell membrane by directly affecting the membrane lipid layers which can lead to cellular necrosis and tissue damage (Stocker & Keaney, 2004). Various factors are responsible for increased free radicals generation in tissues such as abnormal mitochondrial function, NADPH activities, iNOS activities, chemical mediators, and industrial toxins (Lobo, Patil, Phatak, & Chandra, 2010; Rahal et al., 2014). Angiotensin II is an endogenous mediator which may regulate mitochondrial function, NADPH activities, and also increase ROS production in cells (Dikalov & Nazarewicz, 2013; Sachse & Wolf, 2007). Previous report suggests that angiotensin II gets overactivated in 2K1C rats, which subsequently develop hypertension and endothelial dysfunction (Yu et al., 2013). Antioxidants have a protective function against free radical‐induced renal and cardiac dysfunction in animal models. The results of this study suggest that amla powder possesses protective action as well as antioxidant action against renal and cardiac damages in 2K1C rats. This result is also supported by previous report showed that amla extract is protective against renal dysfunction and prevents oxidative stress in kidneys (Muthu, Bobby, Sankar, Vickneshwaran, & Jacob, 2018; Yokozawa et al., 2007).

This study demonstrates that some of the parameters associated with oxidative stress increased in the plasma, heart, and kidney of 2K1C rats compared to the control. The most important oxidative stress parameter is malondialdehyde (MDA), which is a lipid peroxidation product. 2K1C rats showed elevated MDA activity in their plasma, kidney, and heart tissue homogenates which is a clear indication of increased lipid peroxidation in tissues. The present study shows that amla powder supplementation in 2K1C rats has decreased MDA formation in plasma and kidney tissues. This outcome is supported by previous research which showed that amla significantly reduced malondialdehyde levels in serum, renal homogenate, and mitochondria of aged rats and high‐fat diet‐fed rats (Muthu et al., 2018; Yokozawa et al., 2007).

On the other hand, nitric oxide (NO) is a vital molecule in the biological system and is considered as a mediator of many physiological functions. It has been increasingly recognized that NO plays a vital role as an origin of free radicals in the pathogenesis of many diseases (Pacher, Beckman, & Liaudet, 2007). NO, and its metabolite peroxynitrite (ONOO^−^) can cross cell membranes and causes nitration of tyrosine and inactivation of biologically important proteins and enzymes (Pacher et al., 2007). Decreasing NO level could be one of the possible ways of reducing oxidative stress which was also supported by earlier studies. Previously, it has been reported that amla can ameliorate peroxynitrite‐mediated nitrosative stress and prevent tissue damage in rats (Damodara Reddy et al., 2010). In this study, the NO level got increased in the plasma of 2K1C rats, which was normalized by amla powder supplementation. This NO lowering effect could be attributed to the amla extract showed inhibitory effect on iNOS and COX‐2 expression and prevented cytokines activation in kidneys (Muthu et al., 2018; Yokozawa et al., 2007).

In addition, a previous study suggests that patients suffering from chronic kidney diseases (CKD) showed increased plasma concentration of advanced protein oxidation products (APOP) (Conti et al., 2019; Mamun et al., 2020; Witko‐Sarsat et al., 1996). These data also suggest that these oxidized proteins by themselves may contribute to the inflammatory process in CKD (Conti et al., 2019). Advanced protein oxidation product (APOP) is an important marker of oxidative stress in tissues (Conti et al., 2019). Due to increased oxidative stress, APOP might be generated through the reaction of plasma proteins with chlorinated free radicals (Ling & Kuo, 2018; Ulfig, Schulz, Müller, Lupilov, & Leichert, 2019). According to the current investigation, 2K1C rats possessed increased APOP level in plasma, heart, and kidney tissues compared to the control ones that was ameliorated by amla powder (Li et al., 2007; Mamun et al., 2020). Prevention of MDA, NO, and APOP formation in the tissues is further evidenced by the restoration of the tissue antioxidant enzyme functions in this study by amla powder supplementation. Antioxidant enzymes such as catalase and SOD activities got decreased in the 2K1C group compared to the control rats (Mamun et al., 2020). However, these depleted antioxidant enzyme activities were restored by amla supplementation in 2K1C rats which suggests that amla can preserve the tissue antioxidant defense. Previous study also support this notions that amla extract is capable of restoring the antioxidant enzymes such as catalase and SOD activities in acute kidney injuries (Tasanarong et al., 2014).

Uric acid and creatinine are considered as markers of kidney dysfunction (Gowda et al., 2010). In this study, the uric acid and creatinine levels were significantly increased in the 2K1C rats compared to the control group. These findings are similar with another previously reported work (Alam, Chowdhury, Jain, Sagor, & Reza, 2015). Amla powder supplementation in 2K1C rats normalized the levels of uric acid and creatinine concentrations in plasma. However, amla supplementation for four months did not alter the kidney function but decreased oxidative stress in uremic patients (Chen, Liou, & Chang, 2009).

Oxidative markers such as MDA, NO, and APOP tend to get increased in the heart of 2K1C rats that indicates the development of free radical‐mediated oxidative stress in heart (Alam et al., 2015). 2K1C rats also displayed increased number of infiltrated inflammatory cells, collagen deposition and fibrosis in both their kidney and the left ventricle of their heart compared to the control group (Mamun et al., 2020). This histological finding is also supported by the increased MPO activities in kidney and heart tissues of 2K1C rats. Oxidative stress and ROS play a crucial role in the development of fibrosis in the left ventricle of the heart (Mamun et al., 2020; Seddon, Looi, & Shah, 2007). As suggested by this study, amla powder supplementation impeded the inflammatory cells infiltration and also reduced the elevated MPO activities in 2K1C rats. Moreover, amla powder supplementation prevented fibrosis in the kidney and left ventricle of the heart of 2K1C rats. Cardioprotective effect of amla was reported previously showed that cardiac function and antioxidants are restored in isoprenaline induced cardiac damage in rats (Ojha et al., 2012). Another report also suggested that in case of pressure overload‐induced cardiac remodeling, amla extract could help in preventing oxidative stress and fibrosis in the heart of rats (Kumar et al., 2017).

Admittedly, amla powder is a rich source of phenolic antioxidants. The ethanol extract of amla possesses high amount of gallic acid, ellagic acid, and catechin hydrate as confirmed by the HPLC analysis. These natural antioxidants are potent scavenger of free radicles and ROS. Ellagic acid exhibited renoprotective activity in sodium arsenite‐induced rats (Mehrzadi et al., 2018) whereas gallic acid showed its effectiveness against chemical‐induced kidney toxicity in rats (Reckziegel et al., 2016) as well as prevented oxidative stress in the heart of spontaneously hypertensive rats (Jin et al., 2017). Moreover, the renal protective activity of amla powder suggested by the present study is further supported by a previous study which exhibited that amla extract may ameliorate the lipid peroxidation and inhibit the rise of inducible nitric oxide synthase (iNOS) and cyclooxygenase‐II (COX‐II) expression in the aorta of aged rats (Yokozawa et al., 2007).

## 5 CONCLUSION

The current study depicted that amla powder supplementation is effective in improving the renal function of 2K1C rats by reducing oxidative stress. Moreover, control rats supplemented with amla powder did not produce any deleterious effect, which has proven its nontoxic and safe behavior. The mechanisms responsible for these beneficial effects appear to be related to the modulation of oxidative stress parameters by the polyphenolic antioxidants present in the powder. Hence, this study congruous with the observations of many previously conducted studies warrants evaluation of the inevitableness and clinical benefit of amla in patients with chronic kidney diseases in a clinical setup.

## CONFLICT OF INTERESTS

None to declare.
